# Targeting Long Non-coding RNA to Therapeutically Regulate Gene Expression in Cancer

**DOI:** 10.1016/j.omtn.2020.07.005

**Published:** 2020-07-10

**Authors:** Da Fu, Yi Shi, Ji-Bin Liu, Ting-Miao Wu, Cheng-You Jia, Hui-Qiong Yang, Dan-Dan Zhang, Xiao-Li Yang, Hui-Min Wang, Yu-Shui Ma

**Affiliations:** 1 Department of Radiology, The Fourth Affiliated Hospital of Anhui Medical University, Hefei 230012, China; 2 Cancer Institute, Nantong Tumor Hospital, Nantong 226631, China; 3 Central Laboratory for Medical Research, Shanghai Tenth People’s Hospital, Tongji University School of Medicine, Shanghai 200072, China

**Keywords:** lncRNA, tumor, gene regulation, diagnostic marker, therapeutic target

## Abstract

Long-chain non-coding RNAs (lncRNAs) are RNA molecules with a length greater than 200 nt and no function of encoding proteins. lncRNAs play a precise regulatory function at different levels of transcription and post-transcription, and they interact with various regulatory factors to regulate gene expression, and then participate in cell growth, differentiation, apoptosis, and other life processes. In recent years, studies have shown that the abnormal expression of lncRNAs is closely related to the occurrence and development of tumors, which is expected to become an effective biomarker in tumor diagnosis. The sequencing analysis of mutations in the whole tumor genome suggests that mutations in non-coding regions may play an important role in the occurrence and development of tumors. Therefore, in-depth study of lncRNAs is helpful to clarify the molecular mechanism of tumor occurrence and development and to provide new targets for tumor diagnosis and treatment. This review introduces the molecular mechanism and clinical application prospect of lncRNAs affecting tumor development from the perspective of gene expression and regulation.

## Graphical Abstract

**Figure:**
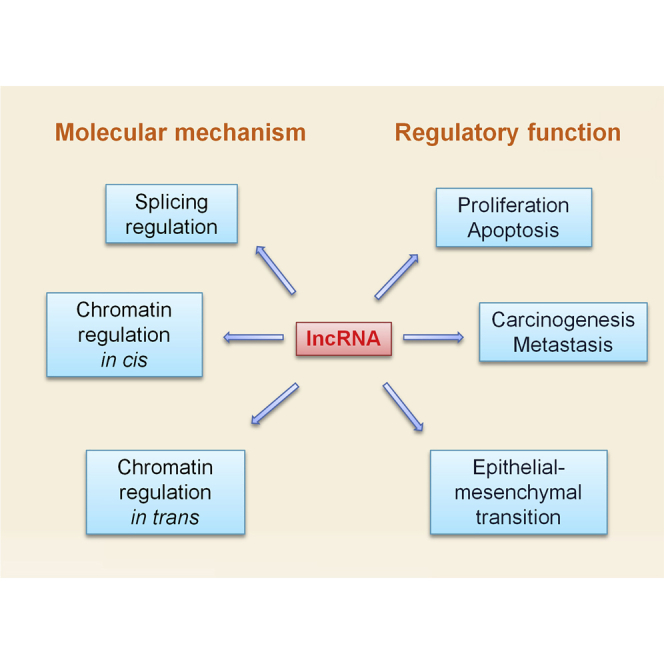

## Teaser

lncRNAs play a precise regulatory function at different levels of transcription and post-transcription and participate in cell growth, differentiation, and apoptosis, and are related to the occurrence and development of tumors, which is expected to become an effective biomarker in tumor diagnosis and treatment.

## Main Text

Cancer is fundamentally a disease of genotype.^1^, ^2^, ^3^ The discovery of protein genetic codon mutations is a breakthrough to understanding the mechanism of these mutations driving tumor development, so as to establish scientific principles for targeted treatment of malignant tumors.^4^, ^5^, ^6^ The human genome project found that less than 3% of the genes in the human genome can be encoded into proteins, which means that the non-coding part has greater potential to drive the characterization of tumors, and there is evidence that if the non-coding region changes, it can affect the expression and regulation of genes, leading to the formation of tumors.^7^, ^8^, ^9^ In recent years, with the deepening of cancer research, the mutation of non-coding genes, the change of epigenetic structures, and the change of genome structures can drive the generation of tumors.^10^, ^11^, ^12^, ^13^ From the point of view of gene expression and the regulation process, the gene expression and regulation of tumor cells are different from those of normal cells, so that they have the ability of infinite proliferation, even invasion and metastasis.^14^

At present, it has been confirmed that long-chain non-coding RNAs (lncRNAs) not only affect the growth and development of embryos, participate in the maintenance of organ and tissue functions, regulate the stability of the immune system, and protect the integrity of telomere structure, but they also are related to the occurrence and development of tumors.^15^, ^16^, ^17^, ^18^ The abnormal expression of lncRNAs often plays a role in promoting or inhibiting tumor development.^19^ lncRNAs participate in the regulation of gene expression and are involved in the biological mechanism of tumor development.^20^ They are expected to become biomarkers for early diagnosis, treatment, and prognosis of tumors.^21^ The expression of lncRNAs in tumor cells has certain specificity.^22^ Its expression level is affected by many factors. Abnormal epigenetic modification is one of the important factors that cause lncRNA expression disorder and disease.^23^ In this review, the biological behavior of lncRNAs in tumors is described, and the role of lncRNAs in tumor occurrence and development, as well as the potential significance of lncRNAs in early clinical diagnosis, prognosis, and treatment target, are explained.

### Discovery of lncRNAs

A lncRNA is a kind of nucleic acid molecule with a length of more than 200 nt, lack of a complete specific open reading frame, and no function of a coding protein.^24^ Generally speaking, lncRNA refers in the narrow sense to lncRNA excluding rRNA, which can be transcribed into more than 200,000 kinds.^25^ Although there are many kinds of lncRNAs, most of the copies in cells are relatively low; some, even with several cells, contain one copy.^26^ Most of the annotated lncRNAs are expressed in specific cell types, and usually at lower levels than the protein coding genes.^27^

**Figure 1:**
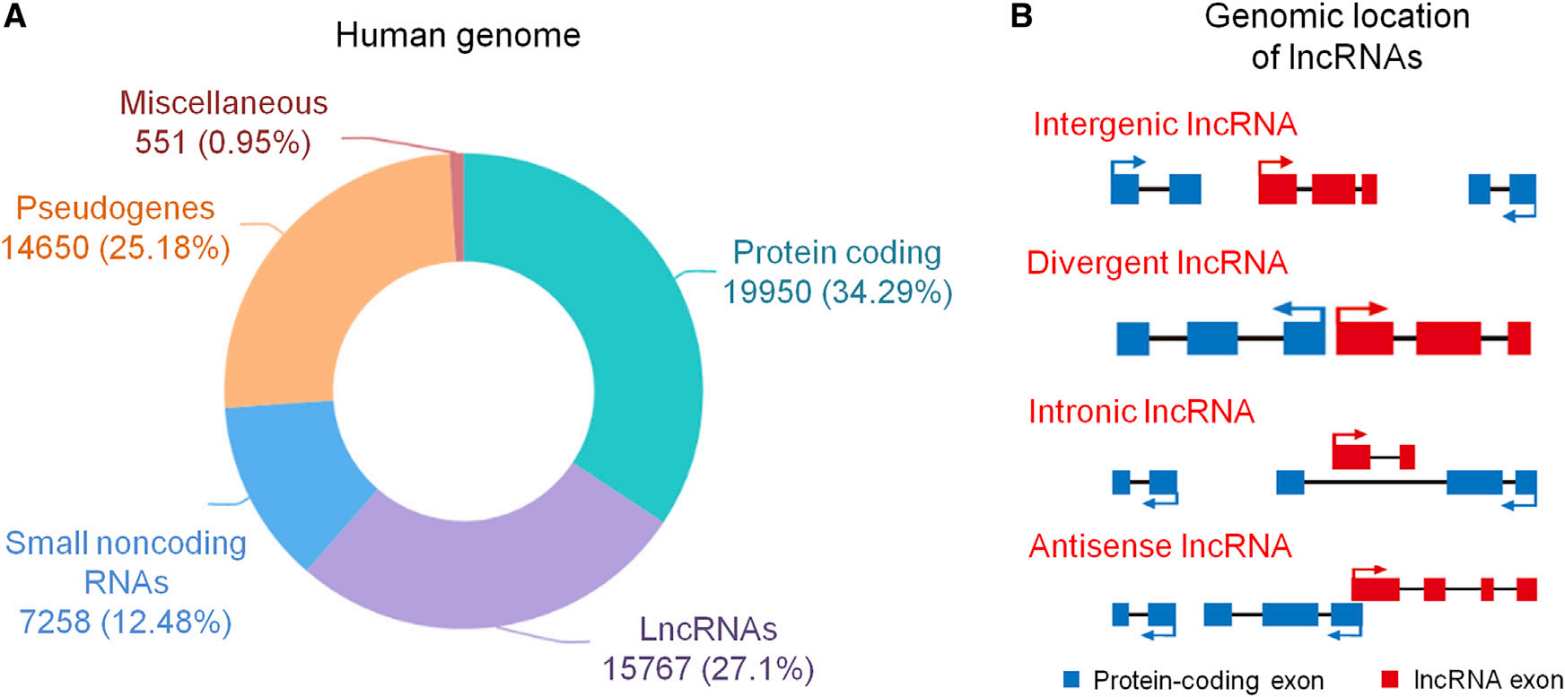
The Abundance and Classification of lncRNAs (A) The abundance of protein-coding and non-protein-coding genes in the human genome. The data represent GENCODE v25 estimates. (B) Classification of lncRNAs based on their genomic location with respect to nearby protein-coding genes.

lncRNAs can be transcribed from the antisense strand, promoter region, intron region, and intergenic region of mRNAs.^28^, ^29^, ^30^ That is, lncRNAs can be transcribed from anywhere in the genome. According to the location of lncRNAs in the genome, lncRNAs can be divided into three categories: long gene non-coding RNAs (lincRNAs), natural antisense transcripts (NATs), and intron lncRNAs.^31^, ^32^, ^33^ In addition to the lincRNAs located between the two protein coding genes, most of the lncRNAs and the adjacent protein coding genes have a certain degree of gene sequence overlap.^29^^,^^34^^,^^35^ For example, intron lncRNAs are transcribed from the intron of the protein coding gene, whereas NATs are transcribed from the opposite (complementary) chain of the protein coding gene.^36^ Antisense lncRNA is especially common in mice.^37^ Up to 72% of the genomic sites show that differential transcription leads to the production of antisense lncRNA^38^ (Figure 1).

Most lncRNAs are transcribed from RNA polymerase II, which are spliced and matured.^39^ Similar to mRNAs, most lncRNAs are blocked, polyadenylated, and spliced.^40^ Their primary structures are poorly conserved, but their secondary structures and splicing patterns are functionally conserved with tissue or cell specificity.^41^ The classification of lncRNAs is based on the idea that RNAs with a base number greater than 200 nt can form various complex high-level structures, which can be distinguished from microRNAs (miRNAs).^42^ In fact, however, when the base number of an RNA is 50–70 nt, some complex structures can be formed.^43^ In addition to the linear structure, lncRNAs also have circular RNAs, which may be affected by the structure. The half-life of circular RNAs is longer and more stable than that of linear lncRNAs.^44^ They also have tissue-specific expression in a specific period, affect the growth and development process, and cause diseases such as tumors.^45^

### lncRNAs Participate in Gene Expression and the Regulation Mechanism

**Figure 2:**
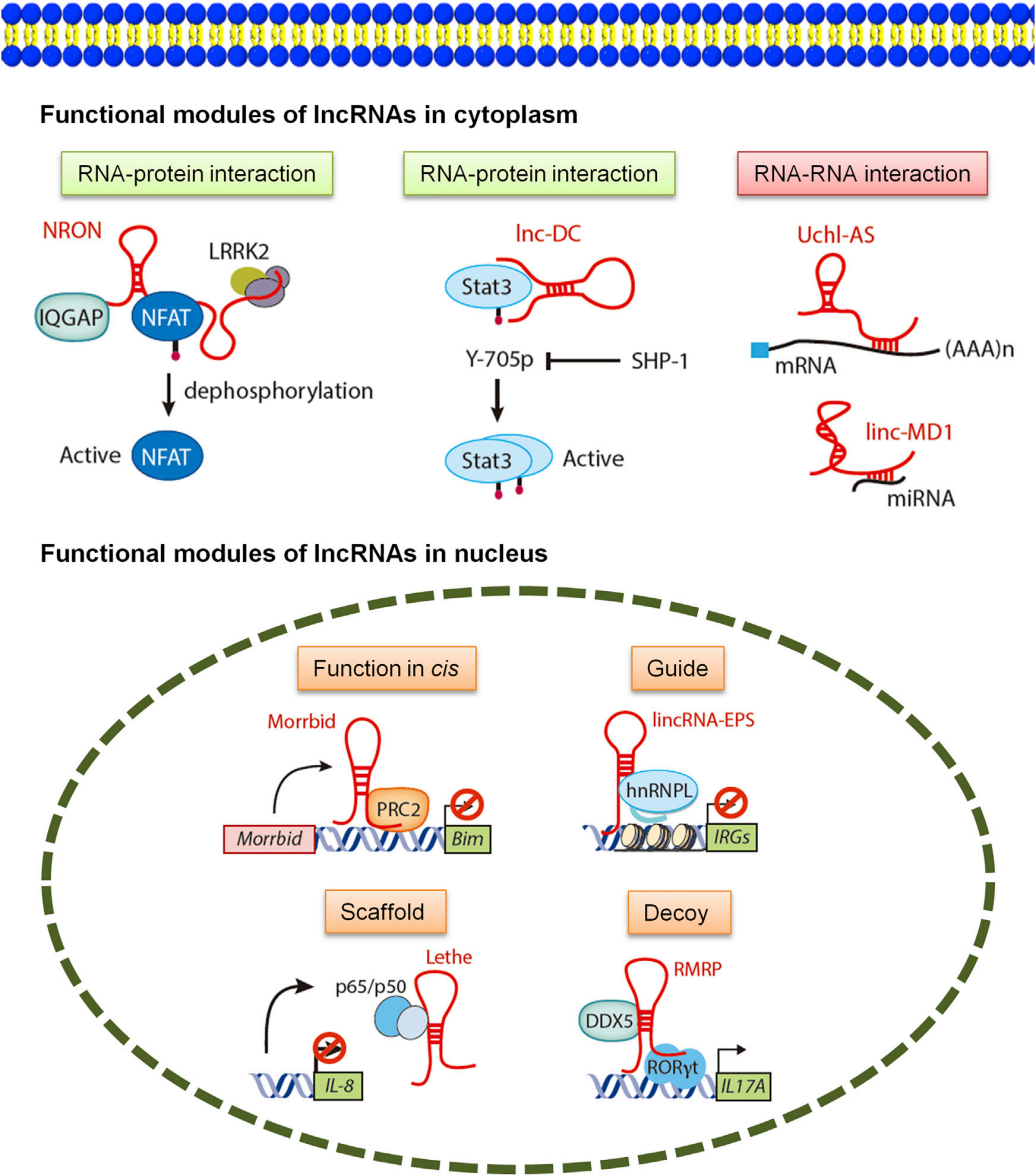
lncRNA Participates in Gene Expression and the Regulation Mechanism lncRNAs mediate their molecular functions through a multitude of mechanisms in the cytoplasm or the nucleus. In the cytoplasm, lncRNAs act through RNA-protein (e.g., NRON and lnc-DC) or RNA-RNA (e.g., Uchl1-AS and linc-MD1) interactions. In the nucleus, lncRNAs can act in *cis* or *trans*. A lncRNA can interact with its protein partner as a guide (e.g., lincRNA-EPS:hnRNPL), scaffold (e.g., RMRP interaction with DDX5), or decoy molecule (e.g., Lethe: NF-κB p65) to mediate its molecular functions.

lncRNAs can interact through RNA-protein, RNA-RNA, or RNA-DNA interactions (Figure 2). lncRNAs can target all levels of gene regulation, including transcription, mRNA stability, and translation. In cytosol, lncRNAs are known to interact with RNA or protein to achieve their molecular functions. For some base pairs of lncRNAs and mRNAs, this interaction can lead to changes in the level of these mRNAs. lincRNA linc-MD1 acts as a competitive endogenous lncRNA to inhibit miR-133.^35^ Antisense lncRNA UCHL1 promotes the translation of UCHL1 mRNA by enhancing the binding of UCHL1 mRNA to the polymer.^22^ On the contrary, some lncRNAs, such as lincRNA-p21, are paired with the target mRNA to inhibit their translation. However, a more common pattern of lncRNA interaction involves interaction with one or more specific proteins. In the nucleus, lncRNAs regulate gene expression through various mechanisms. lncRNAs, as modular guidance and scaffolds for proteins, can recruit proteins or RNAs.^8^ These complexes successively assemble high-order protein/RNA complexes in cells. These proteins cooperate with each other to deposit inhibitory histone markers to silence gene expression of target gene sites. Because lncRNAs guide proteins to specific genomic sites or act as molecular scaffolds to stabilize protein complexes, lncRNAs may also contribute to the functional diversity of DNA-binding proteins.

#### Gene Regulation in the Chromatin Level

The regulation of lncRNA on chromatin mainly affects the structure of chromatin, thus changing gene expression. For example, it interacts with the chromosomal remodeling factor complex, which causes chromatin remodeling, activates or silences gene expression, and affects the occurrence of disease.^46^ By reducing the location of nucleosome in the gene promoter region, the inhibition of gene expression is maintained, leading to disease through epigenetics, and the chromosome can be methylated to inhibit gene expression.^13^ These genes are usually tumor suppressor proteins, because when their expression is inhibited the occurrence of tumors is promoted.

There are two modes of lncRNA regulation at the chromatin level: *cis* regulation and *trans* regulation. *cis* regulation generally refers to when lncRNA plays a regulatory role in the genes adjacent to its transcription region.^47^ The most classic example is X-inactive-specific transcript (XIST). The *Pasteurella* in the nucleus of female mammals is actually a condensed X chromosome, which is formed by the random inactivation of X chromosome due to the metrological compensation effect of cells.^48^ Inactivation of the X chromosome is mainly accomplished by the *cis* function of lncRNA XIST. XIST is a 17- to 20-kb RNA transcribed from the X chromosome. It begins to wrap the X chromosome at the initial stage of inactivation.^49^ By binding with combed protein inhibitor complex 2, H3K27me3 occurs at the position of histone H3K27, which affects the transcription of the chromosome and silences it.^50^ For another example, INK4 protein is a tumor suppressor, and the expression of INK4α/INK4β is also regulated by the antisense chain of its locus, which is transcribed from lncRNA ANRIL (antisense non-coding RNA in the INK4 locus).^51^ lncRNA ANRIL can bind to chromo domain homolog 7 (CBX7). CBX7 is a member of the PRC1 and PRC2 complex. The combination of lncRNA ANRIL and CBX7 makes the histone of this gene site methylated and inhibits the expression of INK4β.^52^ lncRNA ANRIL was initially found to be absent in hereditary tumors of the nervous system. It was found that this RNA was also expressed abnormally in hereditary melanoma of the skin.^53^

**Figure 3:**
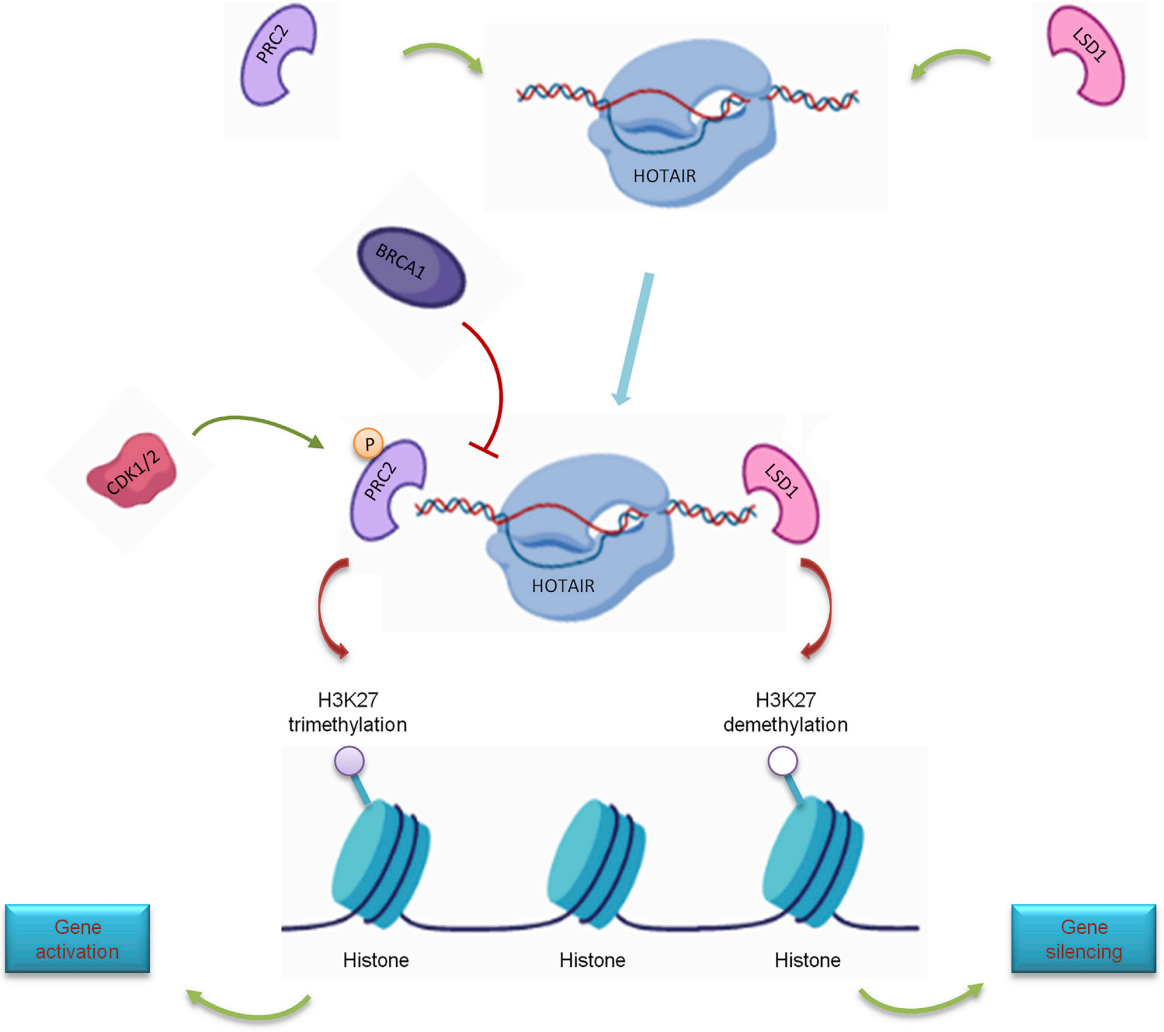
Gene Silencing Mechanism Induced by HOTAIR HOTAIR interacts with PRC2 and LSD1 complexes to recruit them to target genes. PRC2 trimethylated histone H3K27 and LSD1 induced H3K4 demethylation, which silenced the target gene. BRCA1 can compete with HOTAIR to combine PRC2 and inhibit the combination of HOTAIR and PRC2. CDK1/2 mediates the phosphorylation of PRC2 and promotes HOTAIR activation.

The *trans* regulatory RNA is different from the *cis* regulatory RNA, and the RNA and the regulated gene are often located on different chromosomes or both on the same chromosome but far apart.^54^ One of the most famous examples is lncRNA HOTAIR (Hox script antisense RNA). It is a 2.1-kb-long lncRNA transcribed from the HoxC locus on chromosome 12. Its 5′ end can combine with the polycombin complex PRC2, which makes the histone in the HoxD locus region on chromosome 2 undergo epigenetic modification and H3K27me3, resulting in the silencing of HoxD gene expression.^55^ In lung cancer, the expression of HOTAIR is high, which is to make H3K27me3 on the tumor suppressor gene by combining with the PRC2 complex, and then silence the expression.^56^ However, the mechanism of HOTAIR and PRC2 combination is not clear at present, and a thorough understanding of the mechanism of their combination may become a new strategy for cancer treatment in the future^17^ (Figure 3).

Of course, there are also some non-coding RNAs that can be either *cis* regulated or *trans* regulated to play different functions, such as the repetitive repeat-containing RNAs produced by telomere transcription.^57^ Telomere is located at both ends of linear chromatin and, if abnormal, it will lead to aging or cancer and other diseases. Previous studies have pointed out that lncRNA TERRA, together with telomere protein, forms a cap-like structure at the end of chromatin to protect the integrity of chromosomes.^16^^,^^58^^,^^59^ A recent study pointed out that TERRA can not only *cis* act on adjacent telomeres, regulating the activity of telomerase, but also *trans* act on other genes, antagonizing each other with RNA helicase ATRX, thus affecting the expression of its target gene.^60^

#### Transcriptional-Level Regulation

The effect of lncRNA on gene transcription is mainly realized by transcription factors. Mouse retrotransposon VL30 can change the conformation of PSF (polypyrimidine track-binding protein-associated splicing factor), which should be the first non-coding RNA that can directly bind to protein.^61^ PSF protein can inhibit the expression of many proto-oncogenes, thus inhibiting the proliferation and migration of tumor cells; PSF protein has two RNA-binding domains (RBDs) and one DNA-binding domain (DBD) in structure.^62^ Under normal circumstances, the DNA-binding region of the PSF protein can be bound to the promoter of the target gene to inhibit the expression of the target gene. However, when VL30 exists, the RNA-binding region of PSF protein binds to VL30, so as to change the conformation of the PSF protein, so that it can no longer be bound to the promoter of the gene, and the target gene can be expressed.^63^ One of the characteristics of solid tumors is hypoxia. Malignant tumors will accelerate growth and metastasis under a hypoxic environment. Hypoxia inducible factor-1 (HIF-1), which is composed of one α subunit and one β subunit, is the regulator of cells in response to hypoxia.^64^ In a hypoxic environment, HIF-1 will be located in the nucleus, on the promoter of its target gene, and activate the transcription of the target gene.^65^ The target gene of HIF-1 is related to the occurrence and development of tumors, such as glycolysis, energy metabolism, and cell migration, among others.^66^ lncHIFCAR (long non-coding HIF-1α-coactivating RNA) seems to reveal the mechanism of HIF-1 in activating downstream genes. The expression of lncHIFCAR in hypoxia is twice as much as that in the normal condition. It can be combined with HIF-1α to locate on the promoter of the target gene. Meanwhile, HIF-1 and transcription cofactor p300 are recruited to work together to activate the expression of downstream genes.^67^

#### Posttranscriptional-Level Regulation

**Figure 4:**
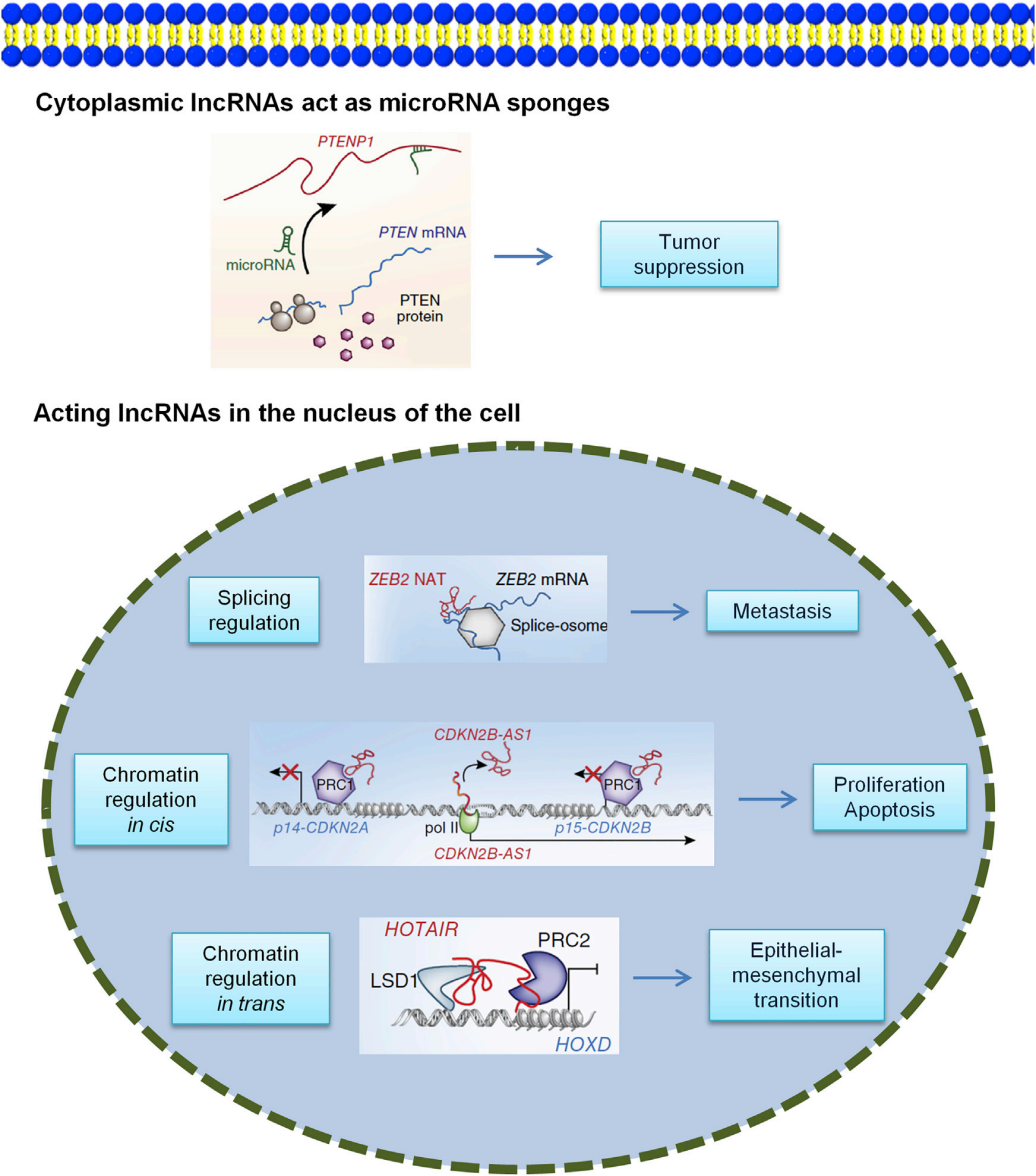
Diverse Mechanisms of Cancer-Related lncRNAs Acting in the nucleus of the cell, some lncRNAs affect the expression of proximally located genes, such as ANRIL (CDKN2B-AS1), which mediates the epigenetic silencing of two genes on the same locus, CDKN2A and CDKN2B, inducing cell proliferation. HOTAIR promotes metastasis in breast cancer by targeting distant genes, such as those in the HOXD cluster, for epigenetic silencing by the PRC2 complex. Other nuclear lncRNAs act post-transcriptionally, such as the NAT of ZEB2 mRNA, ZEB2. The ZEB2 NAT blocks splicing of ZEB2 mRNA, promoting the use of an internal ribosome entry site for translation initiation and delivering high ZEB2 protein levels, which induces epithelial-to-mesenchymal transition. In contrast, a number of cytoplasmic lncRNAs may act as microRNA sponges. For instance PTENP1 binds to microRNAs that otherwise bind to the 3′ untranslated region of PTEN mRNA, reducing its expression and tumor suppressor activity.

The regulation of lncRNA on the post-transcriptional level of genes mainly affects the variable RNA cutting, RNA stability, and translation (Figure 4).

##### Regulation of Variable Cutting

After eukaryotic genes are transcribed into precursor (pre-)mRNA, their introns will be cut off, and their exons will be spliced in different ways, called variable splicing. Therefore, a gene can be encoded into different proteins. More than 95% of the genes in human cells have variable cleavage, which makes the same gene express different proteins in different cells and tissues,^68^ including small nuclear ribonucleoproteins (snRNPs), the serine/arginine-rich (SR) family of nuclear phosphoproteins (SR proteins) and their related egg whites, as well as heterogeneous nuclear ribonucleoproteins (hnRNPs).^69^ Among them, the SR protein family regulates variable shear by its self-phosphorylation state.^70^ Lung cancer-associated transcription 1 (MALAT1) is a lncRNA with a length of about 8.7 kb. It was originally screened from tumor cells of lung cancer patients. It is highly conserved and exists in the paraspots in the nucleus. Early studies suggested that MALAT1 was associated with tumor cell metastasis.^71^ Later, it was found that MALAT1 was involved in variable cutting of genes in normal cells. MALAT1 can locate the phosphorylated SR protein in the paramacula and nucleoplasm, recruit and regulate pre-mRNA, and complete variable shearing; if MALAT1 is knocked out in the cell, the total amount of SR protein in the nucleus will increase, but the phosphorylated SR protein will decrease, and the original shearing site will be changed, so the gene expression will change.^72^

##### Regulation of mRNA Stability

lncRNA can also regulate the stability of mRNA, such as GADD7 (growth-arrested DNA damage-induced gene 7), which is a 754-nt-long lncRNA. It was found that the expression of GADD7 would increase when the cells were damaged by UV rays and in other ways, and the combination of GADD7 and TDP-43 protein (TAR DNA-binding protein 43) would be interrupted, and the combination of the original TDP-43 protein and cyclin-dependent kinase 6 (Cdk6) mRNA would be interrupted, and thus the stability of the mRNA would be reduced and the degradation would be accelerated.^73^

##### Regulation of mRNA Translation

The influence of lncRNAs on mRNA translation is mainly realized by a changing nucleosome, such as lncRNA lincRNA-p21 between genes. The first discovery of lncRNA is that it can regulate p53 protein and inhibit gene expression through the p53 pathway.^74^ Later, it was found that lincRNA-p21 also affects the translation of mRNA. HUR protein is a RNA-binding protein that can bind to mRNA and participate in various cell responses, inflammatory reactions, and tumor formation through the phosphatidylinositol 3-kinase (PI3K)-AKT-nuclear factor κB (NF-κB) signaling pathway.^75^ A recent study showed that the HuR protein is related to the occurrence, invasion, and metastasis of colorectal cancer, gastric cancer, breast cancer, and other tumors.^76^ When there is HuR protein in cells, lincRNA-p21 will become unstable. HuR protein binds to mRNA (such as CTNNB1 mRNA and Jun mRNA) so that ribosomes can smoothly bind to this mRNA and facilitate translation.^77^ If there is no HuR protein in cells, lincRNA-p21 will become stable and increase in number, and then bind to mRNA through base complementary pairing, so as to bind to ribosomes. Site reduction inhibited the translation of this mRNA.^78^ The lncRNAs that play a regulatory role in the process of translation are called translational regulatory lncRNAs (treRNAs), which were first discovered through bioinformatics.^79^ In clinical breast cancer samples with lymph node metastasis, treRNA overexpression promotes tumor cell metastasis and invasion.^80^

The epithelial-mesenchymal transition (EMT) is one of the markers of malignant tumor metastasis. Low expression of E-cadherin can induce the EMT. Although the mRNA level of calmodulin has not changed in malignant tumor cells, the expression of calmodulin is significantly lower than that in normal cells, because the translation-regulated lncRNA affects the translation of calmodulin.^81^ lincRNA-p21 affects the translation of mRNA through complementary base pairing between RNA and RNA, which is totally different from the mechanism of translation-regulated lncRNA.^82^

#### Other Regulatory Roles

Some lncRNAs have more than one regulatory role. For example, XIST is not only involved in the regulation of X chromosome inactivation. Recent studies have found that it can also interact with miRNA and affect the formation of tumors.^83^ As mentioned earlier, lncRNA lincRNA-p21 can not only affect the translation of mRNA, but it also interacts with the heterogeneous protein K in the nucleus, *cis* regulates the expression of p21 protein, and affects the cell cycle change.^84^ In addition to transcriptional regulation of the sense chain, some lncRNAs, which are transcribed from the antisense chain of the locus, have their own functions in other aspects. The tumor suppressor DIRAS3 is related to the occurrence and development of breast cancer and ovarian cancer.^85^ An antisense RNA, lncRNA GNG12-AS1, transcribed from its locus is closely related to tumor metastasis and invasion at the transcription level and post-transcription level, respectively.^86^ At the transcription level, knockdown of the first exon of lncRNA GNG12-AS1 will reduce the transcription level and increase the expression of DIRAS3, thus regulating the cell cycle and inhibiting tumor development.^87^ At the post-transcriptional level, knockdown of the seventh exon of lncRNA GNG12-AS1 will not affect the transcription of the RNA or the expression of DIRAS3, but at this time, the amount of lncRNA GNG12-AS1 will decrease. The epithelial-mesenchymal transformation is enhanced, and the cells will undergo metastasis and invasion.^88^

### Biological Role of lncRNAs in Tumorigenesis and Development

**Figure 5:**
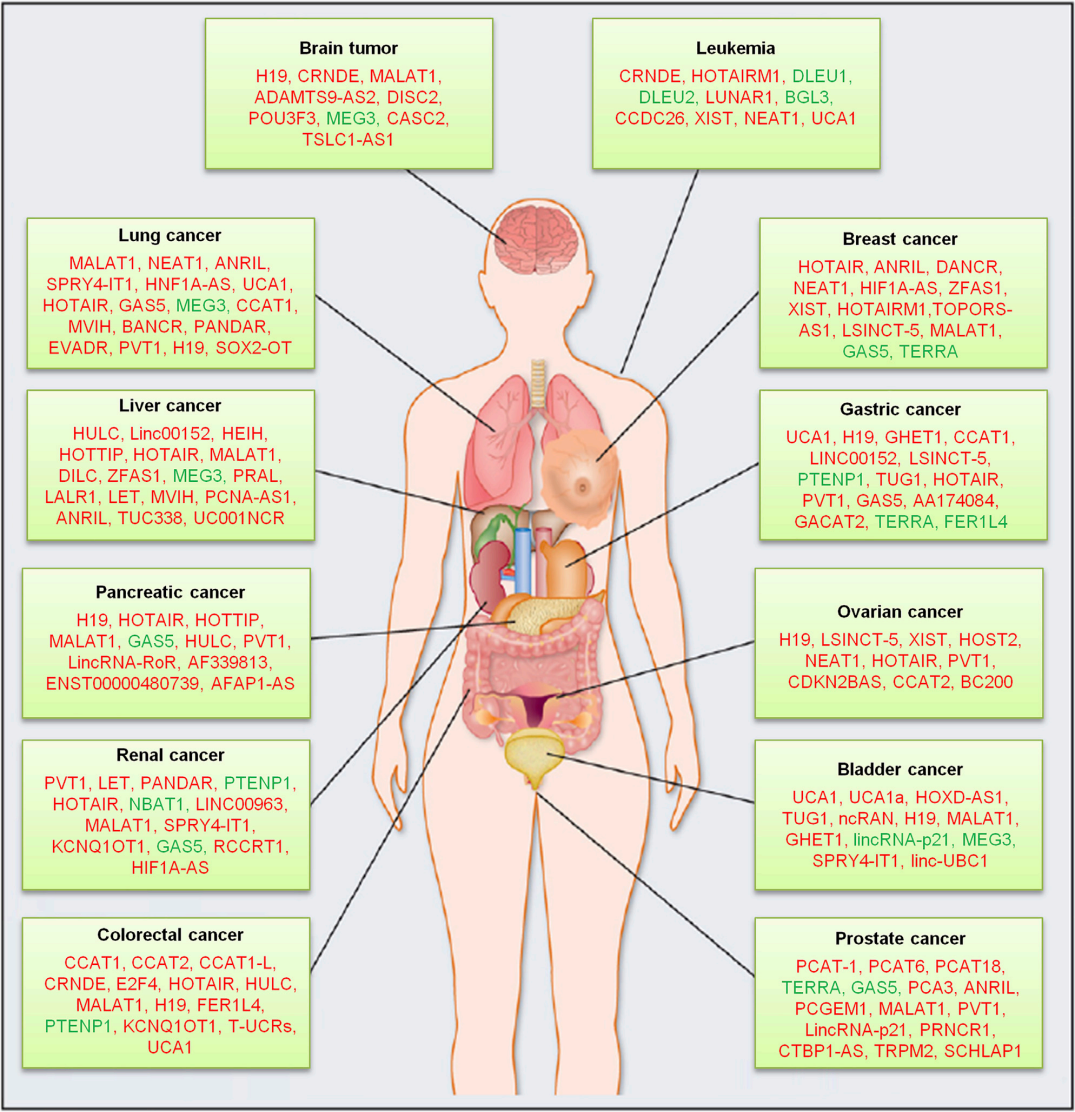
lncRNAs Associated with Various Types of Cancer `Genome-wide association studies of tumor samples have identified a large number of lncRNAs associated with various types of cancer. Alterations in lncRNA expression and their mutations promote tumorigenesis and metastasis. lncRNAs may exhibit tumor-suppressive (green) and tumor-promoting (red) functions. Because of their genome-wide expression patterns in a variety of tissues and their tissue-specific expression characteristics, lncRNAs hold strong promise as novel biomarkers and therapeutic targets for cancer.

At first, it was thought that lncRNAs were the “noise” of genome transcription and the byproduct of RNA polymerase II transcription, which had no biological function.^89^ In recent years, more and more studies have shown that lncRNAs are widely involved in DNA methylation, histone modification, chromatin remodeling, and other biological processes *in vivo*, which can directly interact with transcription factors, functional RNA molecules, and chromatin remodeling modifiers, and regulate the expression of target genes at the epigenetic, transcribed, and post-transcribed levels.^90^, ^91^, ^92^, ^93^ *In vivo*, lncRNAs are mainly used as signaling molecules, bait molecules, guiding molecules, and scaffold molecules to perform biological functions.^94^ In addition, some lncRNAs have diversity in the mode of action and can participate in gene expression regulation in a variety of ways at the same time.^95^ lncRNAs are widely involved in the physiological and pathological processes of the body, play an important role in the occurrence and development of tumors, and have guiding significance in the diagnosis and treatment of diseases (Figure 5).

#### lncRNAs and the Growth of Tumor Cells

Tumor cells can secrete some growth factors to promote their growth.^96^ In T cell lymphocytic acute leukemia, Notch-1 protein activates the transcription of lncRNA LUNAR1, and the combination of the two enhances the expression of insulin-like growth factor 1 and its signaling pathway, and it promotes the growth of tumor cells.^97^ There is a base region lacking transcription activity in the 8q24 region of the human chromosome.^98^ In many malignant tumor cells, the copy number of DNA in this region is increased abnormally, accompanied by the rapid amplification of the proto-oncogene Myc in this region.^99^, ^100^, ^101^ Now there is evidence that lncRNA can participate in Myc oncogenesis. Some lncRNAs regulated by Myc transcription can also cause cell cycle changes and cancer cell proliferation.^102^

In human Burkitt’s lymphoma, the PVT1 gene located in this chromosome region was heterozygous.^103^ The product of the PVT1 gene is a lncRNA PVT1, which is homologous with mouse transcripts. In the mouse model of Myc tumor formation, the amplification of the Myc gene alone is not enough to promote tumor formation. Only when multiple genes including Myc and PVT1 are amplified at the same time can tumors occur.^104^ The lncRNA PCGEM1 specifically expressed in prostate tissue is also located in the 8q24 region of the chromosome, which can bind with Myc protein, enhance the transcription of downstream genes by Myc, and promote the proliferation of prostate cancer cells.^105^

#### lncRNAs and Apoptosis of Tumor Cells

The occurrence and development of tumors are not only due to the rapid proliferation of cells, but are also related to the decline of cell mortality.^106^ In malignant tumor cells, apoptosis is inhibited to ensure the rapid growth of tumor cells. p53 protein, as a tumor suppressor, plays an important role in cell monitoring.^107^ Once the cell is damaged, p53 protein can change the cell cycle or induce apoptosis by repairing DNA.^108^ There was early evidence that p53 protein can bind to a variety of RNAs. Now it has been confirmed that many lncRNAs when combined with p53 can regulate apoptosis through the p53 pathway.^109^ For example, lncRNA PANDA is induced by DNA damage and regulated by p53, and it can combine with transcription factor NF-YA so that it can no longer promote the expression of apoptosis-promoting factors, thus inhibiting cell apoptosis.^110^ Under the environmental pressure of lack of nutrition, tumor cells have growth advantages compared with normal cells.^111^ When the nutrition is insufficient or the growth factor is reduced in the environment, it will induce the formation of lncRNA GAS5, which competently binds to the glucocorticoid receptor with the glucocorticoid response element, thus inhibiting cell apoptosis.^112^ In epithelial ovarian cancer, the expression of GAS5 is lower than that in the adjacent tissues, and GAS5 inhibits DDP resistance and tumor progression of epithelial ovarian cancer via the GAS5-E2F4-PARP1-mitogen-activated protein kinase (MAPK) axis.^113^ Therefore, inhibiting the expression of GAS5 in breast cancer cells can improve the their survival rate in a barren environment, suggesting that increasing the expression of GAS5 can be used to treat breast cancer.

#### lncRNAs and Metastasis of Tumor Cells

Many tumor-related lncRNAs can regulate the invasion and metastasis of tumor cells.^114^, ^115^, ^116^ There is evidence that in tumor cells, MALAT1 can affect the genes of the cell differentiation and the tumor metastasis signaling pathway through variable shear. Knockdown of MALAT1 resulted in increased adhesion and decreased migration of tumor cells.^117^ Most cancer-related deaths are related to tumor cell migration induced by transforming growth factor β (TGF-β).^118^ In hepatoma cells, TGF-β can activate the expression of lncRNA ATB, promote the EMT transformation of cells, and acquire the invasion ability and metastasis.^119^ In breast cancer cells, lncRNA BCAR4 induced by chemokines can combine with transcription factors SNIP1 and PNUTS to respond to CCL21, activate the atypical GLI2 signaling pathway in cells, and promote tumor cell migration.^120^

#### lncRNAs and Chromosome Stability of Tumor Cells

lncRNAs can also be involved in maintaining chromosome stability. Most cancer cells are characterized by unstable chromosome numbers.^121^ p53 protein can interact with a variety of lncRNAs, maintain cell chromosome stability, and regulate cell fate.^122^ Similarly, p53 protein can be regulated by lncRNAs. When the cell DNA is damaged, it will induce the formation of lncRNA DINO, which can directly bind with p53 protein and promote the transcription of the target gene downstream of p53, and determine the cell fate.^123^ The genomic instability caused by the deletion of p53 may make tumor cells accumulate more cancer drivers, thus accelerating carcinogenesis, tumor metastasis, and drug resistance.^124^ In 2016, NORAD, the lncRNA transcribed from the NORAD gene, was found to help cells maintain the normal number of chromosomes. The lncRNA NORAD can control the separation of chromosomes by the protein PUMILIO in the interphase of cell division, so as to maintain the stability of chromosome number.^125^

### The Application of lncRNAs in Tumor Diagnosis and Treatment

The expression of lncRNAs is tissue-specific and has an important impact on the occurrence and development of tumors.^44^^,^^126^ Therefore, some lncRNAs with expression characteristics can be used as clinical diagnosis markers and treatment targets, such as high expression of lncHIFCAR in tumor cells of patients with oral cancer, which can be used as clinical detection markers and treatment targets for the disease.^127^ In patients with gastric cancer, lncRNA AA174084 is in a downregulated state, so it can be used as a marker for the early diagnosis of gastric cancer;^128^ in patients with colorectal cancer, if lncRNA CCAT1 and CCAT2 are highly expressed, it means that the survival rate and recurrence rate of the disease are low, so it can also be used as a clinical examination test marker.^129^ In 2017, lncRNAs were found in exosomes.^130^, ^131^, ^132^, ^133^ In normal cells, these lncRNAs can interact with RNA-binding proteins to neutralize their own effects. In tumor cells, the expression of lncRNAs in exosomes increases, which promotes the occurrence of tumors.^134^

### Outlook

In the process of gene expression, RNA is involved in almost all aspects, and non-coding RNA controls the fate of cells. Even with the occurrence and development of tumors, lncRNA is also inextricably linked with it. However, for many tumors, although we can find some specific high expression or low expression, or even no expression, through high-throughput sequencing, we still do not understand the molecular mechanism thoroughly, and thus we need to do further research in clinical diagnosis and treatment. Generally speaking, although the number of lncRNAs is huge, the mechanism of lncRNA is not clear, and there are some lncRNAs with more than one function, so there is still a large gap in the field of lncRNAs to be understood.

## Author Contributions

Y.-S.M. and D.F. designed and supervised research. All authors interpreted the data and contributed to the final version of the manuscript.

## Conflicts of Interest

The authors declare no competing interests.
